# Anti-LL37 Antibodies Are Present in Psoriatic Arthritis (PsA) Patients: New Biomarkers in PsA

**DOI:** 10.3389/fimmu.2018.01936

**Published:** 2018-09-12

**Authors:** Loredana Frasca, Raffaella Palazzo, Maria S. Chimenti, Stefano Alivernini, Barbara Tolusso, Laura Bui, Elisabetta Botti, Alessandro Giunta, Luca Bianchi, Luca Petricca, Simone E. Auteri, Francesca Spadaro, Giulia L. Fonti, Mario Falchi, Antonella Evangelista, Barbara Marinari, Immacolata Pietraforte, Francesca R. Spinelli, Tania Colasanti, Cristiano Alessandri, Fabrizio Conti, Elisa Gremese, Antonio Costanzo, Guido Valesini, Roberto Perricone, Roberto Lande

**Affiliations:** ^1^Istituto Superiore di Sanità, National Center for Drug Research and Evaluation, Rome, Italy; ^2^Rheumatology, Allergology and Clinical Immunology, University of Rome Tor Vergata, Rome, Italy; ^3^Division of Rheumatology, Fondazione Policlinico Universitario A. Gemelli, IRCCS, Rome, Italy; ^4^Division of Rheumatology, Università Cattolica del Sacro Cuore, Rome, Italy; ^5^Institute of Pathology, Fondazione Policlinico Universitario A. Gemelli, Rome, Italy; ^6^Dermatology Unit, Department of Systems Medicine, University of Rome Tor Vergata, Rome, Italy; ^7^Rheumatology Unit, Department of Internal Medicine and Medical Specialties, Sapienza University of Rome, Rome, Italy; ^8^Confocal Microscopy Unit, Core Facilities, Istituto Superiore di Sanità, Rome, Italy; ^9^National AIDS Center, Istituto Superiore di Sanità, Rome, Italy; ^10^Department of Oncology and Molecular Medicine, Istituto Superiore di Sanità, Rome, Italy; ^11^Skin Pathology Lab, Humanitas Clinical and Research Center, Milan, Italy

**Keywords:** Psoriatic arthritis, psoriasis, LL37, autoantibodies complement, neutrophils

## Abstract

Psoriatic arthritis (PsA) is a chronic inflammatory arthritis associated with psoriasis. A third of psoriatic patients develop PsA *via* unknown mechanisms. No reliable diagnostic markers are available for PsA, or prognostic biomarkers for PsA development in psoriasis. We previously uncovered a pro-inflammatory role for cathelicidin LL37 in lesional psoriasis skin. LL37 binds nucleic acids and stimulates plasmacytoid/myeloid dendritic cells (pDC, mDCs) to secrete type I interferon (IFN-I) and pro-inflammatory factors. LL37 becomes an autoantigen for psoriatic Th1-Th17/CD8 T cells. Anti-LL37 antibodies were detected in systemic lupus erythematosus, an autoimmune disease characterized by neutrophil-extracellular-traps release (NETosis) in target organs. LL37 can be substrate of irreversible post-translational modifications, citrullination or carbamylation, linked to neutrophil activity. Here we analyzed inflammatory factors, included LL37, in PsA and psoriasis plasma and PsA synovial fluids (SF)/biopsies. We show that LL37 (as a product of infiltrating neutrophils) and autoantibodies to LL37 are elevated in PsA, but not OA SF. Anti-LL37 antibodies correlate with clinical inflammatory markers. Anti-carbamylated/citrullinated-LL37 antibodies are present in PsA SF/plasma and, at lower extent, in psoriasis plasma, but not in controls. Plasma anti-carbamylated-LL37 antibodies correlate with PsA (DAS44) but not psoriasis (PASI) disease activity. Ectopic lymphoid structures, and deposition of immunoglobulin-(Ig)G-complexes (IC) co-localizing with infiltrating neutrophils, are observed in PsA and not OA synovial tissues (ST). Activated complement (C5a, C9), GM-CSF and IFN-I are up-regulated in PsA and not OA synovia and in PsA and psoriasis plasma but not in HD. C9 and GM-CSF levels in PsA SF correlate with clinical inflammatory markers and DAS44 (C9) and with anti-carbamylated/citrullinated-LL37 antibodies (GM-CSF and IFN-I). Thus, we uncover a role for LL37 as a novel PsA autoantibody target and correlation studies suggest participation of anti-LL37 antibodies to PsA pathogenesis. Notably, plasma antibodies to carbamylated-LL37, which correlate with DAS44, suggest their use as new disease activity markers. GM-CSF and complement C5a and C9 elevation may be responsible for autoantigens release by neutrophils and their modification, fueling inflammation and autoreactivity establishment. Finally, targeting GM-CSF, C5a, C9 can be beneficial in PsA.

## Introduction

Psoriasis is a systemic inflammatory and autoimmune skin disease of unclear etiology affecting 1–3% of individuals worldwide (1). Red and scaly plaques caused by the hyperproliferation of skin epithelial cells characterize plaque psoriasis, the most common form (1). Notably, up to 30% of psoriasis patients develop Psoriatic arthritis (PsA) (2), a type of spondyloarthritis characterized by enthesitis, dactylitis peripheral arthritis and axial involvement. Diagnostic criteria of PsA are primarily clinical and based on the Classification Criteria for Psoriatic Arthritis (CASPAR) and include evidence of psoriasis, absence of rheumatoid arthritis (AR), and the exclusion of other seronegative arthritis (3). Thus, no reliable serological markers are available to identify PsA, as for RA. Histologically, PsA is characterized by lining layer hyperplasia, innate immune cell activation, T and B-lymphocytes infiltrated synovial tissues, and synovial angiogenesis (4). As for RA, a role of B cells and autoantibodies has been suggested in the pathogenesis of PsA (5). Indeed, although the presence of rheumatoid factor (RF) and anti-citrullinated peptide antibodies (ACPA) are characteristic of RA, and uncommon in PsA (6), the detection of ectopic lymphoid structures in PsA synovia (7) suggests production of antibodies against local autoantigens. Notably, anti-carbamylated peptide autoantibodies (anti-CarP Abs) have been recently identified not only in RA (8), but also PsA plasma (9). Currently, no reliable diagnostic biomarkers distinguish PsA from psoriasis and no prognostic markers are available to predict the development of PsA in psoriasis patients.

Cationic antimicrobial peptides (AMP) including the cathelicidin LL37 are aberrantly produced by psoriatic keratinocytes and released by degranulating neutrophils or during neutrophil extracellular trap formation (NETosis) (10–12). LL37 has the ability to bind nucleic acids and induce the production of pro-inflammatory cytokines and type I interferon (IFN-I), by plasmacytoid dendritic cells (pDCs) and myeloid dendritic cells (mDCs) via TLR7/8/9 triggering (13, 14). Moreover, LL37 has been recognized as self-antigen for psoriatic autoreactive T-cells that are detected in circulation or in lesional skin (15). Both CD4 and CD8 T lymphocytes respond to LL37 and LL37-specific CD4 T cells belong to Th1/Th17 sub-populations. Interestingly, in a systemic autoimmune disease such as systemic lupus erythematosus (SLE), where LL37-DNA complexes are highly released during NETosis, LL37 becomes the target of pathogenic autoantibodies (12, 16). Although neutrophils can infiltrate psoriatic skin (17), and a study suggests that NETosis is possibly occurring in skin lesions (18), whether LL37 becomes the target of autoantibodies in psoriasis patients has not been investigated. Notably, there are few reports that show expression of LL37 in inflamed synovia (19). However, whether and how LL37 plays a role as autoantigen in PsA is still unknown.

In this picture, with the idea to: (1) identify the pathogenic players in PsA, (2) discriminate the immunological pathways that are in common or are distinct between psoriasis and PsA, and (3) identify new disease activity markers for PsA, we have investigated the presence of LL37 and related autoantibodies in PsA and psoriasis patients and analyzed their correlations with clinical parameters and inflammatory factors.

## Results

### LL37 and autoantibodies to LL37 are present in PsA synovia

**Figure 1:**
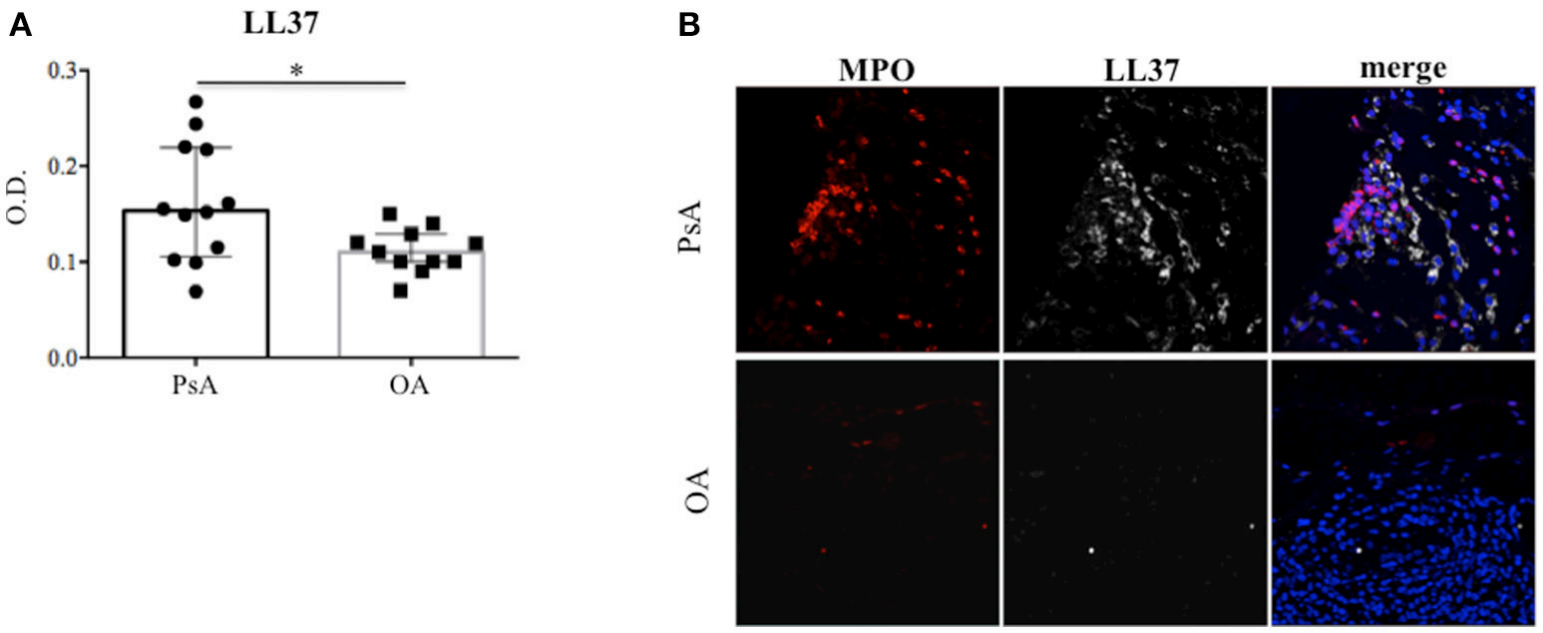
LL37 is expressed in synovial compartment of PsA. **(A)** LL37 was measured by ELISA in synovial fluids of PsA (*N* = 12) and control OA patients (*N* = 11), and LL37 levels are shown as median with Interquartile Range (IQR). P-value is calculated by two-tailed Mann-Whitney U test *p < 0.05. **(B)** Confocal microscopy images of synovial tissues of PsA and OA patients stained for myeloperoxidase (MPO; red), LL37 (gray) (original magnification 63x). For PsA, 1 representative staining of 7 patients is shown. For OA, 1 representative staining of 4 patients is shown.

**Figure 2:**
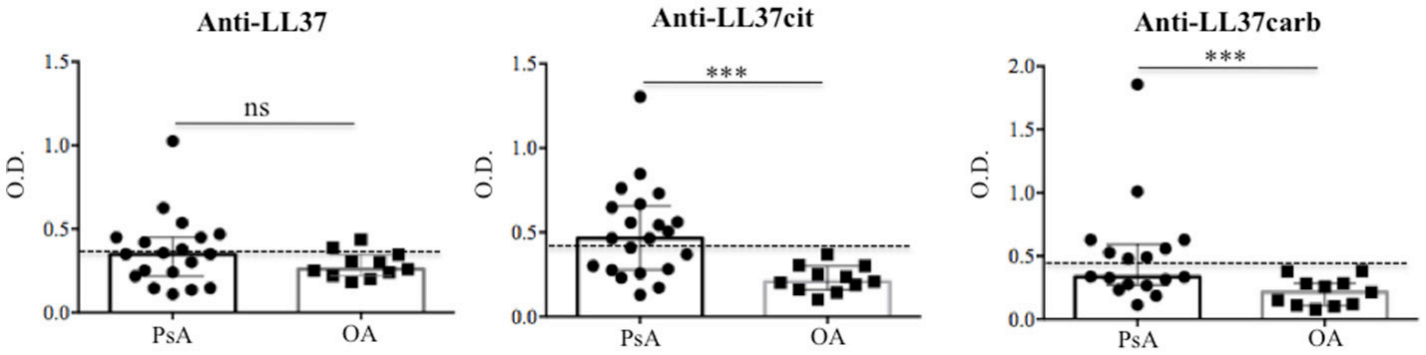
Anti-LL37 antibodies are present in synovial fluids of PsA. Synovial fluids of PsA and OA (*N* = 11) patients were analyzed by ELISA for the presence of anti-LL37 (PsA, *N* = 19), anti-LL37cit (PsA, *N* = 21) and anti-LL37carb (PsA, *N* = 17). Antibody levels are shown as median with Interquartile Range (IQR). *P*-value is calculated by two-tailed Mann-Whitney U test, ***p < 0.0001. The mean+2 SD (standard deviation) of OA antibody reactivity to native LL37 or modified LL37 was used as cut-off (dotted line).

In order to investigate the putative role of LL37 in PsA, we firstly analyzed whether LL37 was measurable in the synovial compartments. As shown in Figure 1A, higher levels of LL37 were detected in the synovial fluids (SF) of PsA (median: 0.153, IQR: 0.114) compared to control osteo-arthritis (OA) patients (median: 0.1, IQR: 0.036), *p* = 0.031, by ELISA assay. Laser scanner confocal microscopy of synovial biopsies from patients affected by early PsA showed a consistent staining for LL37, coupled to high staining for myeloperoxidase (MPO), the typical marker of neutrophils, in the lining and sub-lining areas of the synovial membranes (Figure 1B). This suggested that LL37 was present in PsA synovial tissues (ST) as the product of neutrophils, although a contribution of other cells might not be excluded. In contrast, only occasionally neutrophils and LL37 positivity were detectable in control OA synovia (Figure 1B). Neutrophil-derived antimicrobial peptides, including LL37, have been shown to become target of circulating autoantibodies in SLE patients (12, 16) and, to date, one study reported ectopic lymphoid tissues in PsA synovia (7). Thus we assessed the presence of anti-LL37 antibodies in SF of PsA and control OA patients. Although the antibody levels did not reach a statistical significance between PsA (median: 0.348, IQR: 0.233) and OA SF (median: 0.26, IQR: 0.126), *p* = 0.28, by setting a cut-off (as in Figure 1A) we found that autoantibodies to LL37 were present in 7 out of 19 PsA SF (37%) (Figure 2).

It is reported that LL37 can become a substrate for post-translational modifications such as citrullination and carbamylation (20, 21). Of note, carbamylated proteins can be the targets of autoantibodies in PsA (9). In this context, we wondered whether we could also detect antibody reactivity toward either citrullinated (LL37cit) or carbamylated (LL37carb) LL37 or both in PsA SF.

**Figure 3:**
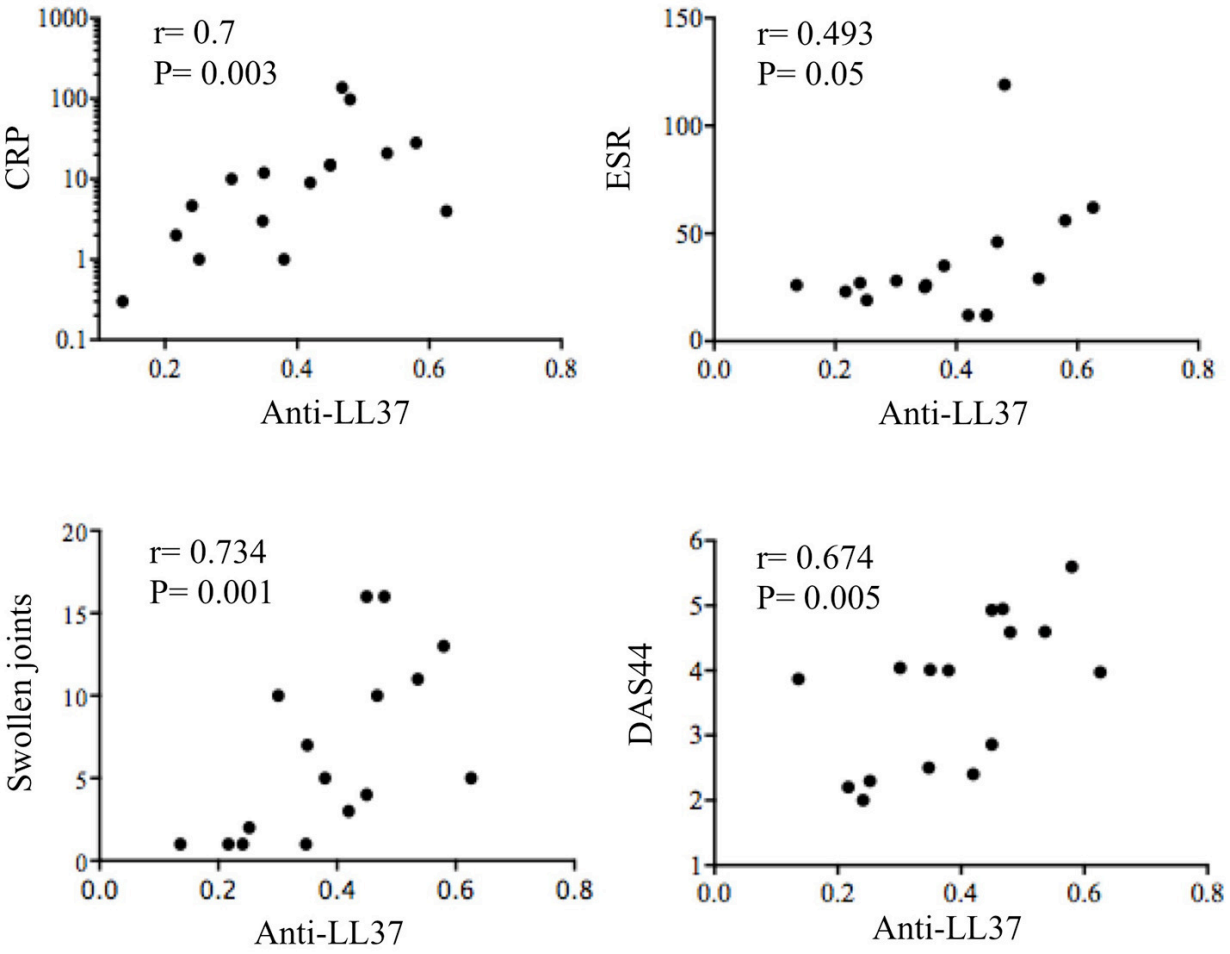
Anti-LL37 levels in SF PsA correlate with disease activity and inflammation markers. SF PsA levels of anti-LL37 Abs (*N* = 16) measured by ELISA as shown in Figure 2 were correlated with Disease Activity Score (DAS44), C reactive Protein (CRP), Eritrocyte Sedimentation Rate (ESR) and number of swollen joints. The correlation between the two variables was assessed by Spearman correlation coefficient.

Interestingly, anti-LL37cit antibodies were higher in PsA SF (median: 0.464, IQR: 0.379; 12 out of 21: 57%) than in OA SF (median: 0.207, IQR: 0.14), *p* = 0.0008; similarly, anti-LL37carb antibodies were higher in PsA SF (median: 0.335, IQR: 0.321; 8 out of 17: 47%) than in OA SF (median: 0.214, IQR: 0.174), *p* = 0.004, (Figure 2). Of note, there was no significant correlation between anti-LL37 antibody reactivity to native and either citrullinated or carbamylated LL37, suggesting that anti-LL37cit and anti-LL37carb antibody reactivity is probably not due to cross-reactivity but is likely specifically directed toward modified LL37. This indicates that pathways of protein modification are likely to be activated in PsA synovia. Next, we investigated possible correlation between anti-LL37-autoantibody reactivity and clinical parameters such as inflammatory markers and disease activity (DAS44). We found that levels of anti-LL37 antibodies to native protein correlated with C-reactive protein (CRP), erythrocyte sedimentation rate (ESR), swollen joints and DAS44 (Figure 3). In contrast, no significant correlation was observed between the same clinical data and antibodies to LL37cit or LL37carb. Altogether, these results suggest that LL37 can become an autoantigen in PsA and the antibody response to LL37 in SF may represent a marker of inflammation and disease activity.

### Anti-LL37 autoantibodies are detected in circulation of PsA and psoriasis patients

**Figure 4:**
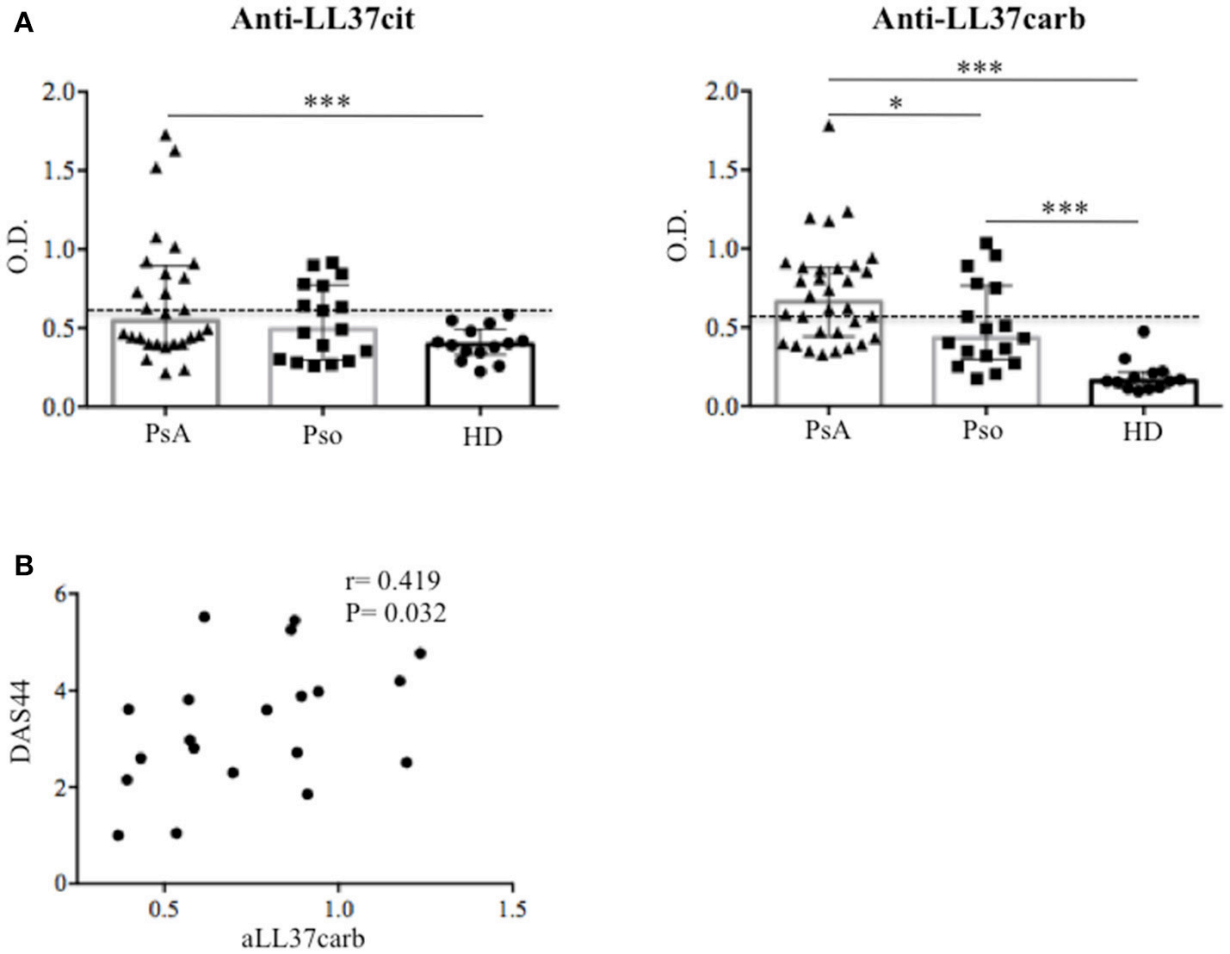
Autoantibody reactivity to citrullinated and carbamylated LL37 in plasma of PsA and Pso patients. **(A)** Levels of anti-LL37cit (*N* = 29) and anti-LL37carb (*N* = 32) were measured in plasma of PsA, Pso (without PsA, *N* = 17) and healthy donors (HD, *N* = 14) by ELISA. Antibody levels are shown as median with Interquartile Range (IQR). *P*-value is calculated by two-tailed Mann-Whitney U test, *p < 0.05, ***p < 0.0001. The mean of HD antibody reactivity+2 SD was used as cut-off (dotted line). **(B)** Levels of PsA anti-LL37carb were correlated with Disease Activity Score (DAS44) by Spearman correlation coefficient and the significant *P* value by two-tailed Mann-Whitney U test.

In order to establish whether the presence of anti-LL37 antibody reactivity was a local phenomenon or whether the same reactivity was systemically detectable, we assessed plasma of PsA, psoriasis and control healthy donors (HD), for antibodies to native LL37, LL37cit, and LL37carb by ELISA. Anti-LL37 antibody reactivity to the unmodified peptide was below the cut-off both in PsA and psoriasis (not shown). However, anti-LL37cit antibodies were higher in PsA plasma (median: 0.544, IQR: 0.495; 11 out of 29: 32%) than in HD plasma (median: 0.394; IQR: 0.16), *p* = 0.007. Anti-LL37carb antibodies were higher in PsA (median: 0.66, IQR: 0.439; 18 out of 32: 52%) than in HD plasma (median: 0.158, IQR: 0.099), *p* = 0.0001 (Figure 4A). No statistical significance was observed between anti-LL37cit plasma levels of PsA vs. psoriasis plasma (median: 0.49, IQR: 0.478), *p* = 0.3. In contrast, the anti-LL37carb levels of PsA plasma were significantly higher also when compared to psoriasis plasma (median: 0.43, IQR: 0.47), *p* = 0.02. By setting a cut-off we found that 5 out of 17 (29%) psoriasis patients were positive for anti-LL37cit or anti-LL37carb antibody reactivity (Figure 4A). However, levels of anti-LL37cit of psoriasis plasma were not significantly higher compared to HD plasma (*p* = 0.15); in contrast, levels of anti-LL37carb antibodies of psoriasis plasma were significantly higher compared to levels of HD plasma (*p* = 0.0001). Moreover, we observed a significant positive correlation between plasma anti-LL37carb antibodies and disease activity (DAS44) in PsA (Figure 4B), in line with recent evidences reporting presence of antibodies to carbamylated proteins in plasma of PsA patients (9). No correlations were apparent between plasma anti-LL37carb or anti-LL37cit antibodies and clinical inflammatory parameters in PsA. Antibody reactivity to LL37cit and LL37carb did not show any correlation with psoriasis activity score index (PASI) in psoriasis patients. These data suggest that measurement of serum levels of anti-LL37carb, but not anti-LL37cit antibodies, may be used as a marker of disease activity in PsA and not in psoriasis.

### PsA synovia show IgG-immune complex deposition

**Figure 5:**
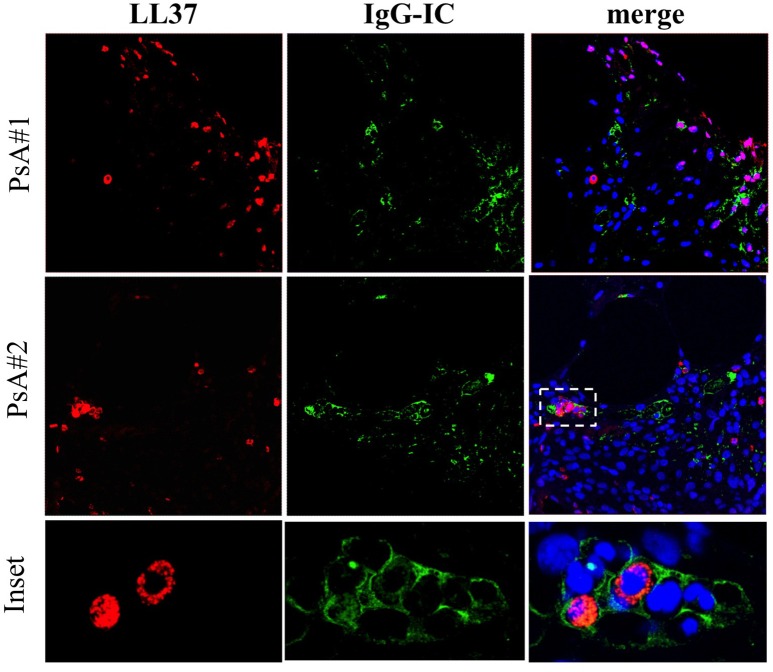
IgG**-**immune complexes deposition and colocalization with neutrophil-derived LL37 in synovia of PsA. Confocal microscopy images of synovial tissues of two PsA patients stained for LL37 (red), IgG-IC (green) (original magnification, 63x). The dotted white line in PsA#2 indicates the inset of the corresponding picture of the bottom panel. Data are representative of 7 PsA patients.

The deposition of immune complexes (IC) in inflamed tissues is a pathogenic mechanism described in various autoimmune diseases (22, 23). The presence of autoantibodies in synovial fluids and plasma of PsA patients prompted us to investigate the deposition of IgG-IC in ST of PsA, and in OA as control, by confocal microscopy. We visualized the presence of IC in ST of PsA (Figure 5) but not of control OA patients (not shown). Interestingly, some of the IC^+^-synovial cells were infiltrating neutrophils, as demonstrated by a consistent co-localization of IgG and LL37 staining in cells with the typical polymorfo-nuclear shape (Figure 5, inset). IgG staining was detectable also in LL37-negative cells, probably macrophages and/or fibroblasts. These results indicate that IgG are detectable in ST of early PsA patients and that they may represent deposit of immune complexes in PsA synovia; these IgG seemed to include ANCA-like antibodies, suggesting that, among the various antibody specificities, anti-LL37 antibodies may be present and target neutrophils.

### Ectopic germinal center-like structures are detected in synovia of early PsA

**Figure 6:**
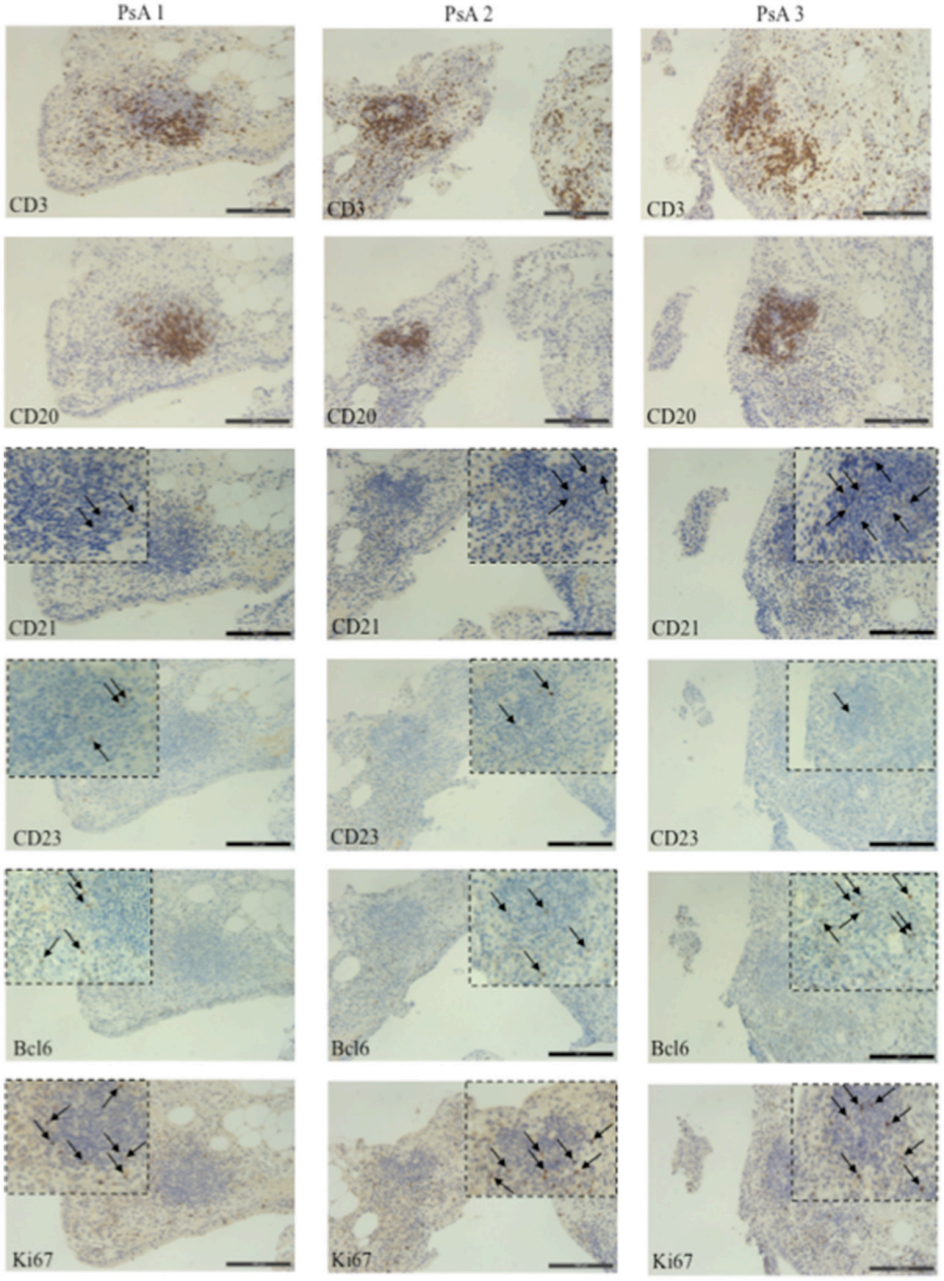
Germinal Center-like structures in synovial tissues of PsA patients. IHC for CD3, CD20, CD21, CD23, Bcl6, and Ki67 (all DAB, brown) of synovial tissue from three early, naive to DMARDs treatment PsA patients (original magnification, 20x). The black arrows indicate a positive staining for CD21, CD23, Ki67, and Bcl6. The dotted black line indicates the insert of the corresponding picture at 40x magnification.

Ectopic lymphoid structures resembling germinal centers have been characterized in chronically inflamed tissues in RA and their presence was associated with an antigen driven B cell response (24–27). To date, only one study addressed the presence of ectopic lymphoid structures in PsA ST (7). We addressed whether lymphoid-like structures might develop in PsA ST already at early disease stage and tried to more finely characterize them: we selected PsA patients with early stage of psoriatic arthritis and naïve to any disease modifying anti-rheumatic drugs (DMARD). We analyzed, by immunohistochemistry (IHC), the presence of infiltrating T and B lymphocytes and presence of CD21^+^ or CD23^+^ follicular dendritic cells (FDC), together with the expression of the activation/proliferation marker Ki67 and Bcl6, the latter expressed by GC B cells and involved in the development of CD4^+^ T-follicular helper cells during the GC reaction [Tfh; (28–30)]. As shown in Figure 6, we observed the presence of CD3^+^ T cells and CD20^+^ B cells aggregates in the ST of early naïve PsA that were associated to CD21^+^ and CD23^+^ FDC. The presence of a lower staining for CD23^+^ cells as compared to that for CD21^+^ cells appears in line with other works reporting that the complement receptor CD21 represents a more stable phenotypic marker of FDC (31, 32). Moreover, we found a wide expression of the cell proliferation marker Ki67 and presence of Bcl6^+^ cells located in the T and B cell aggregates. Altogether these data indicate the presence of organized extra-nodal lymphoid structures in PsA synovia and suggest that such structures can develop at very early stages of disease. Indeed, anti-LL37 antibody reactivity was detectable in PsA synovial fluids and plasma of individuals with early stage disease.

### Activated complement and GM-CSF are present in PsA synovial compartments and in circulation

The complement system is activated at tissue site where there is antibody deposition, as described in systemic lupus erythematosus (SLE) and RA (33, 34). Complement C5a has been described either as a potent granulocyte chemoattractant (35) or as a priming factor for neutrophil degranulation (36). Moreover C5a, in the presence of GM-CSF, activates neutrophils to undergo NETosis accompanied by release of granules (37). Other complement members, such as C5b-C9, are involved in the activation of citrullination pathways. This is mediated by the peptidyl arginine deiminase (PAD) activation via Ca^++^ influx, following formation of the Membrane Attack Complex (MAC) that induces pore formation in leukocytes (38). Since we observed presence of IgG in the PsA synovial compartment and antibody reactivity to both native and citrullinated LL37, we assessed the content of C5a, C9, and GM-CSF in SF of PsA and control OA patients and, for comparison, in plasma of PsA and psoriasis patients.

**Figure 7:**
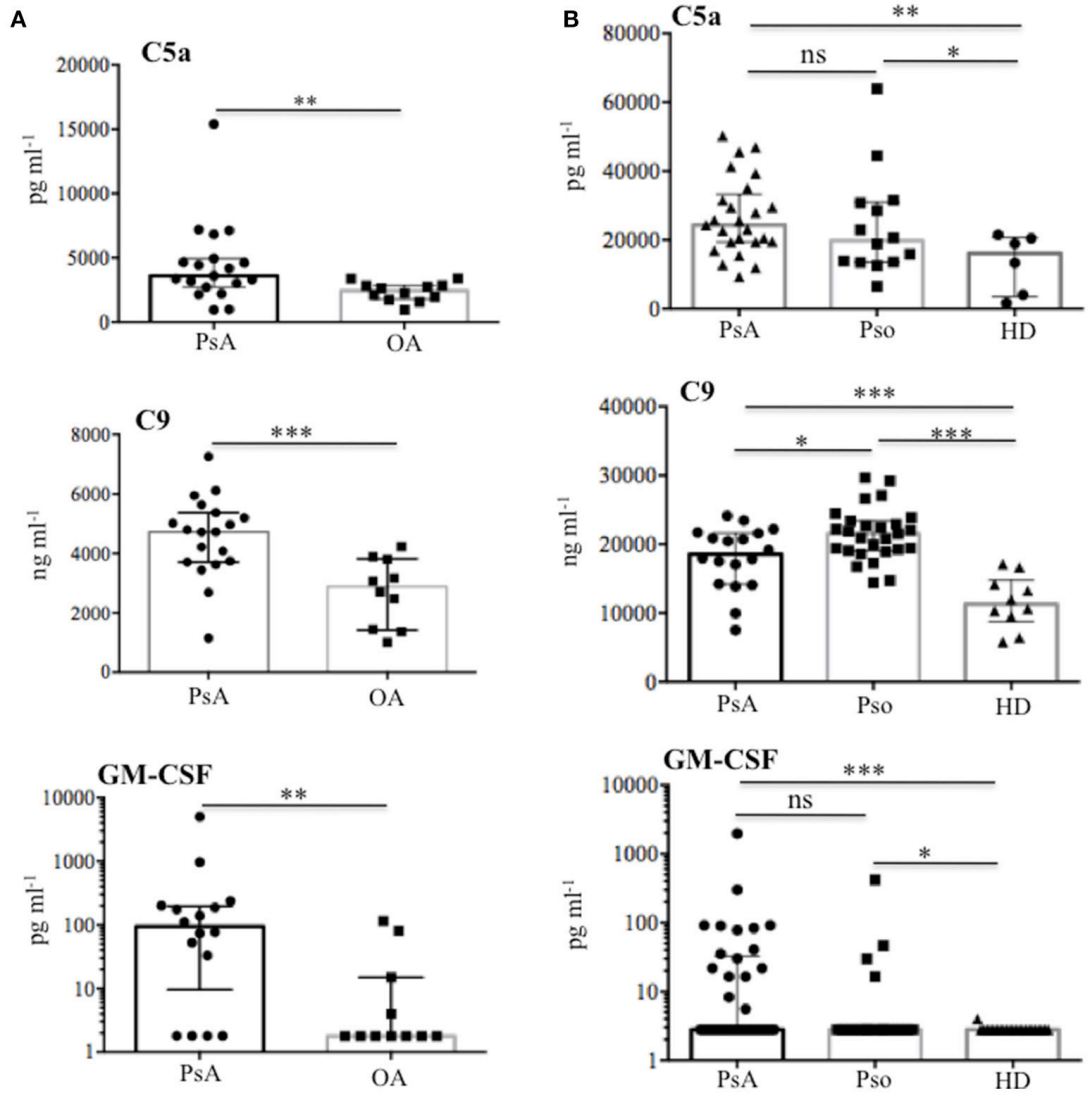
Complement C5a, C9, and GM-CSF in SF and plasma of PsA and Pso patients. **(A)** Content of C5a (*N* = 19), C9 (*N* = 19), and GM-CSF (*N* = 21) were measured by ELISA in SF of PsA and OA (*N* = 12). *P*-value is calculated by two-tailed Mann-Whitney U test, *p < 0.05, **p < 0.01, ***p < 0.0001. **(B)** Plasma levels of C5a in PsA (*N* = 25) and Pso (without PsA, *N* = 14), PsA C9 (*N* = 18), and Pso (*N* = 25), PsA GM-CSF (*N* = 25) and Pso (*N* = 16) were assessed by ELISA. All data in **(A)** and **(B)** are shown as as median with Interquartile Range (IQR). *P*-value is calculated by two-tailed Mann-Whitney U test, *p < 0.05, **p < 0.01, ***p < 0.001.

**Figure 8:**
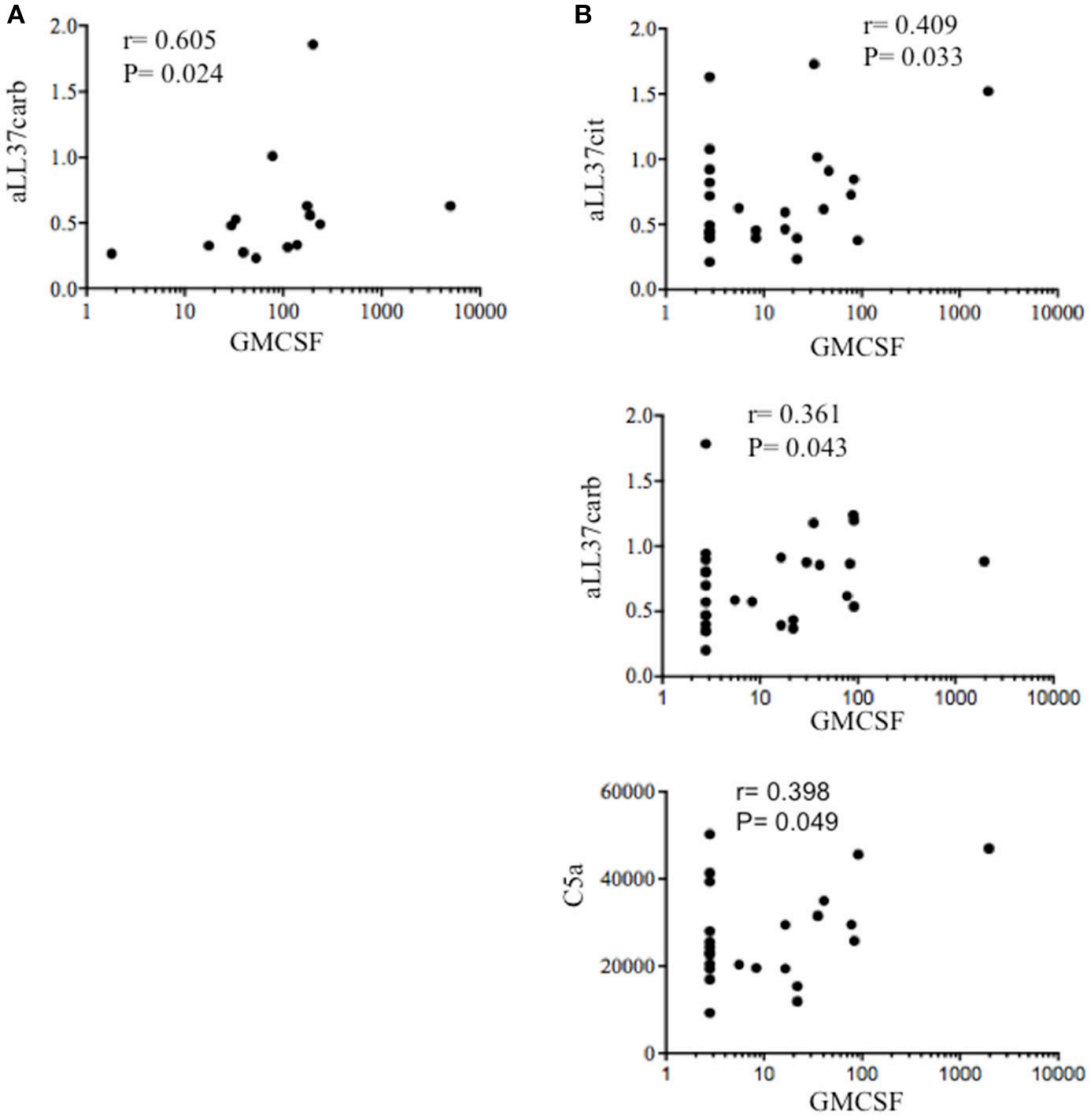
GM-CSF correlates with complement and anti-LL37 antibodies in PsA. **(A)** Spearman correlation analysis was performed between levels of GM-CSF and anti-LL37carb in SF PsA (*N* = 17). **(B)** Spearman correlation analysis between GM-CSF and C5a, anti-LL37cit, and anti-LL37carb in plasma of PsA patients (*N* = 23).

**Figure 9:**
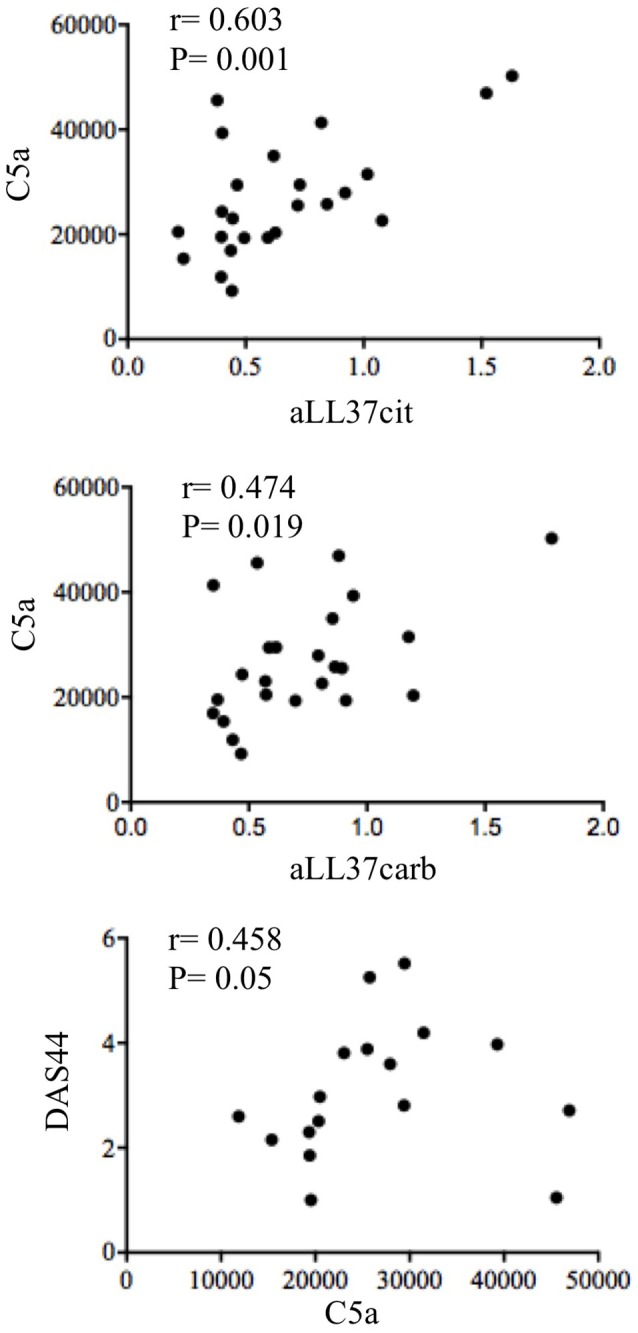
Circulating C5a correlates with anti-LL37 antibodies and disease activity. Spearman correlation analysis was performed between levels of C5a with anti-LL37cit (*N* = 24), anti-LL37carb (*N* = 24) and DAS44 in plasma of PsA (*N* = 17).

**Figure 10:**
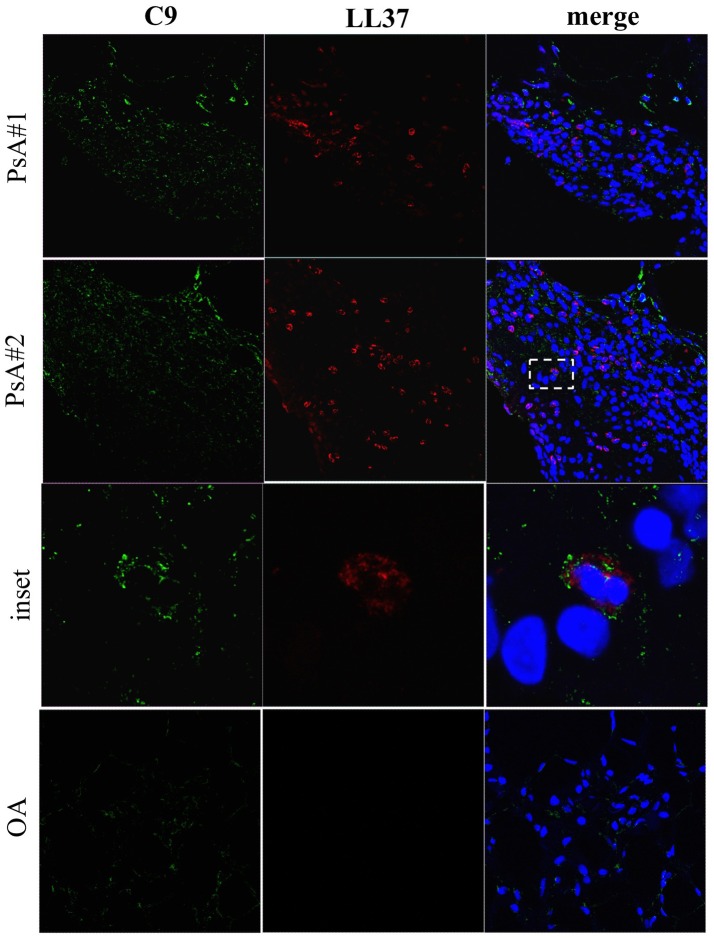
C9 staining in synovial tissues of PsA. Confocal microscopy images of synovial tissues of two PsA and one OA patients stained for LL37 (red) and C9 (green) (original magnification, 63x). The dotted white line in PsA#2 indicates the inset of the corresponding picture of the lower panel. Data are representative of 7 PsA patients and 4 OA subjects.

As shown in Figure 7A, we found a significantly higher concentration of C5a in PsA SF (median: 3590, IQR: 2234) as compared to OA SF (median: 2426, IQR: 1065), *p* = 0.007; C9 was also higher in PsA SF (median: 4727, IQR: 1665) than OA SF (median: 2884, IQR: 2399), *p* = 0.001. GM-CSF was up-regulated in PsA SF (median: 94.45, IQR: 187) as compared to OA SF (median: 1.79, IQR: 13.21), *p* = 0.001. Moreover, C5a in PsA plasma was higher (median: 24309, IQR: 13863) than in HD plasma (median: 16107, IQR: 17310), *p* = 0.013; C9 in PsA plasma was higher (median: 18542, IQR: 7425) than in HD (median: 11292, IQR: 6023), *p* = 0.0009; GM-CSF in PsA plasma was higher (median: 2.79, IQR: 29.87) than in HD plasma (median: 2.79, IQR: 0), *p* = 0.001 (Figure 7B). Of note, the same factors were elevated in the plasma of psoriasis patients. Indeed, the concentration of C5a (median: 19741, IQR: 17432), *p* = 0.05, C9 (median: 21526, IQR: 17432), *p* = 0.0001 and GM-CSF (median: 2.79, IQR: 0), *p* = 0.05, were higher in psoriasis as compared to HD plasma, although the elevation of GM-CSF and C5a were at the limit of the statistical significance (Figure 7B). By comparing PsA plasma to psoriasis plasma, only C9 levels were significantly higher in psoriasis than in PsA, *p* = 0.02. C9 levels in synovial fluids, but not in plasma, strongly correlated with disease activity (DAS44; *r* = 0.773, *P* = 0.001; *N* = 16) and various inflammatory parameters: CRP (*r* = 0.734, *P* = 0.008), swollen joints (*r* = 0.726, *P* = 0.01) and tender joints (*r* = 0.729, *P* = 0.009). GM-CSF levels correlated with both anti-LL37cit and anti-LL37carb antibodies in PsA plasma (Figure 8B) and with anti-LL37carb (but not with anti-LL37cit) in PsA SF (Figure 8A). Moreover, GM-CSF correlated with C5a in PsA plasma (Figure 8B). Finally, C5a levels significantly correlated with both anti-LL37cit and anti-LL37carb antibodies in plasma (Figure 9), and showed a correlation with DAS44 in PsA, although this correlation was at the limit of the statistical significance (*P* = 0.05; Figure 9). Of note, we observed no correlation between circulating C9 or C5a levels and PASI in psoriasis patients. In line with the presence of C9 in PsA SF, we have visualized an abundant presence of C9 in ST of PsA as compared to OA patients by confocal microscopy (Figure 10). C9 staining partially colocalized with neutrophils (Figure 10).

These results and the relative correlation analyses suggest a participation of activated complement and GM-CSF to antibody reactivity development in PsA and suggest a link between presence of these factors and posttranslational modification of self-proteins in PsA (namely citrullination and, in particular, carbamylation).

### An IFN-I signature is present in SF of PsA patients

Given that immune complexes can induce IFNα (13–15) and an IFN-I signature is present in several immune mediated diseases (SLE, RA), we assessed the presence of IFNα in the synovial compartment of PsA and OA patients.

**Figure 11:**
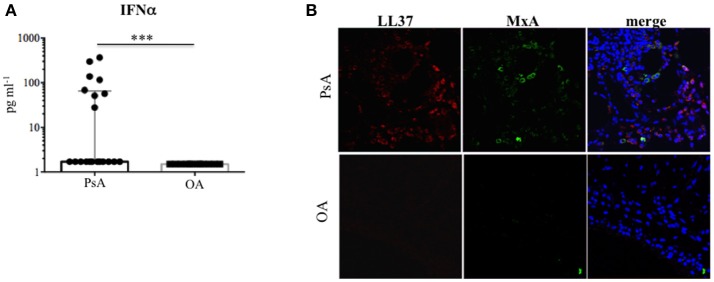
Type I IFN in the synovial compartment of PsA. **(A)** IFNa was measured by ELISA in SF of PsA (*N* = 20) and OA (*N* = 12) subjects. Data are shown as median with Interquartile Range (IQR). *P*-value is calculated by two-tailed Mann-Whitney U test ***p < 0.001. **(B)** Confocal microscopy images of synovial tissues of one PsA and one OA patient stained for LL37 (red) and MxA (green) (original magnification, 63x). Data are representative of 7 PsA patients and 4 OA subjects.

IFNα was measurable by ELISA assay in 8 out of 20 (35%) PsA SF (median: 1.7, IQR: 63.5) and not in OA SF (median: 1.5, IQR: 0), *p* = 0.0001 (Figure 11A). To confirm this, we stained PsA and OA ST with a specific surrogate marker of local IFN-I production, the protein MxA (39, 40). MxA staining was extensively found in all PsA ST analyzed (Figure 11B), but not in OA ST. MxA staining was distributed either on neutrophils or other unidentified cell types. Although MxA was in part distributed in the vicinity of LL37, the levels of SF IFNα did not correlate with LL37 or anti-LL37 antibodies, with disease activity and/or clinical inflammation markers.

Altogether these findings suggest IFN-I pathways are activated in PsA synovia, and, since ST analyzed by confocal microscopy belong to early diagnosed PsA, an IFN-I signature can represent an early event in disease onset.

## Discussion

PsA develops in 20–30% of psoriasis patients, and skin lesions of psoriasis develop 5–10 years before PsA (41). However the mechanisms linking psoriasis to the development of PsA are still elusive. More generally, no reliable serological markers are available for PsA diagnosis as compared to RA, and PsA pathogenesis is not elucidated.

Results of this study suggest novel factors, some of which are also implicated in psoriasis pathogenesis (13, 15, 36), included the antimicrobial peptide LL37, that seem at work in the pathogenesis of PsA and are potential biomarkers of inflammation/disease status. First of all, we found that LL37 is highly up regulated in SF of PsA patients as compared to control OA. A previous study showed the presence of mRNA for LL37 in inflamed synovial membranes, however analysis of the protein on PsA synovial tissues and fluids were not addressed (42). We found that LL37 is abundantly expressed in PsA ST by confocal microscopy. Since the ST included in the present study belong to very early (<8 months from disease diagnosis) PsA patients, naïve to any pharmacological treatment, these findings indicate an early up regulation of LL37 in PsA ST. Second, we show for the first time that LL37 becomes the target of autoantibodies, representing a novel autoantigen in PsA which sustains the idea of PsA as an autoimmune disease (9). Of note, these anti-LL37 antibodies have been found in synovia and plasma of patients with a very early disease (disease duration between 3 and 10 months) suggesting the development of autoimmunity as an early event. Noteworthy, the patient cohort analyzed in this study includes mainly early PsA patients, in that the mean of disease duration is 11 months. Most intriguing, the presence of autoantibodies to native LL37 in SF well correlates with several inflammatory makers (CRP, ESR, swollen joints count) and disease activity (DAS44) in the patients analyzed, suggesting a pathogenic role of anti-LL37 antibodies in PsA. These observations fit with the detection of extra-nodal germinal centers (GC)-like structures in PsA ST, which shows that these lymphoid ectopic structures are present in early and naïve ST of PsA. This suggests that the production of autoantibodies occurring at early stages may start as a local phenomenon. Indeed, we visualized for the first time the presence of rare FDC (CD21^+^ and CD23^+^ cells) together with evidences of cell proliferation (Ki67^+^ cells) and markers of both GC B cells and follicular T helper cells (Bcl6^+^ cells), which suggests that these ectopic lymphoid aggregates can be functional in early PsA. Extranodal lymphoid structures have been identified in other autoimmune or chronic inflammatory diseases such as RA, Sjogren's syndrome, autoimmune thyroid disease, multiple sclerosis or chronic infections (32, 43–45). In these structures lymphocytes are organized and aggregated to form B cell follicles and T cell areas. The functional activity of GC is dependent on the presence of a network of FDC, which retain antigens on their membranes in the form of IC leading to B cell maturation (46). In addition, we show that early synovia also present tissue IgG-IC deposition. Interestingly, IgG staining by confocal microscopy shows co-localization with LL37 in tissue infiltrating neutrophils. These findings suggest an important role of neutrophils as a source of local autoantigens in PsA and may indicate these cells as the target of autoantibody aggression. Neutrophils deliver the content of their granules by degranulation and/or NETosis, phenomena favored by C5a and/or GM-CSF (47, 48) stimulation. Our data show the abundant presence of these factors in SF and also in circulation of PsA patients. Thus C5a and GM-CSF can initiate PsA pathogenesis by attracting and activating neutrophils to deliver their factors by degranulation or in the form of NET (37, 49). NET release of LL37 has been described in SLE where anti-LL37 antibodies are indeed generated (12). Neutrophils have been indicated as a source of autoantigens also in RA (50, 51). The suggestion that C5a and GM-CSF play a role in pathogenic pathways that ultimately lead to autoimmunity via neutrophil activation is supported by the fact that GM-CSF levels in the synovial compartments correlate with autoantibody reactivity. This correlation has been found, in particular, with the presence of anti-LL37 antibodies that react to the post-translational modified versions of LL37 (LL37cit and LL37carb), whereas a good correlation has been also found between anti-native LL37 antibodies and inflammatory parameters in SF. The reason for this discrepancy is unclear at present, and we are not able to explain it completely. However, it is possible that the determination of reactivity to the native or modified LL37 forms may depend on the specific affinity of the tested autoantibodies, as well from the LL37 preparations used in the assay. Crucial can be their degree of citrullination or carbamylation that is high (see Methods) and may not exactly reflect degree of such modifications *in vivo*. Indeed, LL37 carbamylation and citrullination have been observed *in vitro* and not *in vivo* so far (20, 21).

However, the detected reactivity to both citrullinated and carbamylated LL37 is intriguing and deserves further investigation to understand whether reactivity to the modified LL37 is really distinct from reactivity to the native peptide and the site where this autoreactivity is firstly generated. An important finding is that anti-LL37 antibodies were below the detection limit in PsA circulation, whereas antibodies to modified LL37 were consistent, which is difficult to explain. However, the observation that a percentage of PsA patients respond to carbamylated LL37 and, most importantly, that the magnitude of the antibody reactivity significantly correlates with disease activity (DAS44), suggests to use this immunological parameter as a disease marker in PsA, distinct from psoriasis. Indeed, although also psoriasis patients show antibody reactivity to LL37carb, psoriasis activity index (PASI) did not correlate with this type of antibodies, and levels of anti-LL37carb in psoriasis is significantly lower than that observed in PsA plasma. Our data also support previous findings that PsA patients have high levels of circulating antibodies recognizing carbamylated proteins (9).

The importance of post-translational modification of LL37 in PsA in breaking immunological tolerance to native LL37 is not clarified by our data. However, the fact that antibody reactivity against an autoantigen that is the substrate of both citrullination and carbamylation (20, 21) is induced, reinforces the assumption that neutrophils are crucial player. Indeed, we found strong neutrophil infiltrate in ST of PsA, with abundant presence of MPO, whose activity is strictly connected to production of cyanate (52), which, in turn, favors carbamylation of self-proteins during neutrophilic inflammation. Citrullination is another self-protein modification classically ascribed to neutrophils activity, namely NETosis (50). Although it is matter of debate whether citrullination always occur during NETosis, it is also possible to hypothesize that hypercitrullination phenomena may occur due to MAC activation (38). In this regards, our data show a high presence of activated complement in PsA and C9 detection in tissues. Thus, out data suggest that anti-neutrophil cytoplasmic (ANCA)-like antibodies, such as anti-native LL37, can deposit in ST early during the disease course, where they target neutrophils and activate complement: we assume that abundant neutrophil proteins are released and undergo citrullination and/or carbamylation. This phenomenon activates specific autoantibodies, included anti-LL37 antibodies reacting to the modified antigen, which amplifies a pathogenic pernicious loop. The action of LL37 antibodies can also favor the activation of an IFN-I signature in tissues via TLR7/8/9, as shown in SLE (12), given the binding capacity of LL37 for self-nucleic acids. Cells other than pDCs can also contribute the IFN-I signature observed in PsA synovial, via this mechanism. Moreover, some kind of NETosis (included live NETosis; 37, 39) is likely to occur in PsA (our unpublished observations) as in RA (38), and this may also explain the IFN-signature that we have observed (MxA staining in synovial tissues). Levels of IFNα were also elevated in some PsA SF, although IFNα did not correlate with autoantibodies or disease activity. However, this finding lead us to speculate that, in PsA, IFN-I and NET may directly favor the generation of autoantibodies, at least in memory B cells (16).

Finally, our data showing the presence of complement component C9 in ST and peripheral blood of PsA patients are intriguing, since levels of C9, especially in SF, strongly correlate with all disease parameters (clinically relevant inflammatory parameters and disease activity, as DAS44). Thus, these data lend support to the hypothesis that complement activation is an important player in PsA, and its targeting may be beneficial (53).

In conclusion, the data of the present study shed light on possible new players/mechanisms in PsA pathogenesis (LL37, anti-LL37 antibodies, NETosis, self-protein modifications) while reinforcing involvement of previously suspect players (activated complement, carbamylation, GM-CSF) in the disease. We report that, (i) LL37 behaves as a novel B cell autoantigen in PsA, also in its modified form (LL37cit and LL37carb), (ii) pathways of protein carbamylation and citrullination are likely activated in PsA, (iii) degranulating/netting neutrophils are players in the release of the autoantigens (for instance LL37), under the effect of inflammatory factors, such as GM-CSF and C5a. IgG-immune complexes formation and their deposition in PsA ST can fuel this vicious circle and also induce an IFN-I signature (12). Since we have shown through our study that some of the characteristics found in PsA are present also in psoriasis patients without PsA (circulating complement, anti-LL37carb antibodies, GM-CSF), longitudinal follow-up of psoriasis patients with antibody reactivity to LL37 and higher complement and GM-CSF levels may be highly informative about the evolution of psoriasis into a more systemic inflammation that eventually develop to PsA. This may allow the identification of prognostic biomarkers for PsA development in psoriasis patients. Finally, almost all patients with PsA have psoriasis and we have shown that LL37 is a CD4 and CD8 T cell autoantigen in at least 46% of psoriasis patients (15). LL37-specific-T cells and their Th1/Th17 secretion pattern correlate with PASI (15). Approximately, the same percentage of PsA patients show CD4 and CD8 T cells proliferating to LL37, which is reasonable (our unpublished data). Whether these PsA T cells exhibit a different phenotype (cytokines secreted, homing receptors and/or T-helper cells markers) that may explain the development of PsA in psoriasis-affected patients in comparison to psoriasis-only patients is under investigation.

## Materials and methods

### Study population

The study included thirty-two Caucasian patients 18 years old or older, rheumatoid factor and ACPA negative, affected by PsA according to the CASPAR classification criteria for Psoriatic Arthritis, and recruited from the Rheumatology Unit of the University of Rome Tor Vergata, Sapienza University of Rome, and the Institute of Rheumatology of Fondazione Policlinico Universitario A. Gemelli IRCCS - Catholic University of the Sacred Hearth of Rome.

Patients were treated with csDMARDs (conventional synthetic Disease-Modifying Anti-Rheumatic drugs) in 43% (14/32) and with bDMARDs (biological-DMARDs) in 12% (4/32). Joint disease activity was measured with the number of tender and swollen joint, the pain-VAS (visual analogue scale) and the GH (global health) and by using the Disease Activity Score 44 (DAS44) with the evaluation of the CRP (C-Reactive Protein) and of the erythrocyte sedimentation rate (ESR). Skin disease activity was assessed using PASI (Psoriasis Area Severity Index). Clinical and demographic data of PsA, Pso, and osteoarthritis (OA) subjects are summarized in Table 1. Chronic plaque psoriasis diagnosis was based on a confirmed diagnosis for at least 6 months before. Criteria of exclusion: pustular, erythrodermic, and/or arthritis form of psoriasis; history of drug-induced psoriasis; clinically significant flare of psoriasis during 12 weeks prior; concurrent or recent use of any biologic or systemic therapy; received non-biologic systemic psoriasis therapy or phototherapy (including psoralen and ultraviolet A, PUVA), ultraviolet B (UVB) within the previous 4 weeks; or had topical treatment within the previous 2 weeks prior.

**Table 1:** Data are expressed as mean ± standard deviation unless otherwise specified; range of possible values are indicated in square brackets.

**Demographic and clinical data**	**PsA patients (*n* = 32)**	**PsO patients (*n* = 24)**	**OA patients (*n* = 12)**
Male sex (N/%)	19 (59)	11 (13)	7 (58)
Age (years)	54.3 ± 11.9	51.1 ± 12.1	45.7 ± 15.7
Pso (N/%)	22 (69)		
PsA disease duration (months)	11 ± 5	0 (0)	
Pso disease duration (months)	20 ± 10	15.3 ± 10	
ESR (mm/h)	16.6 ± 10,1		
CRP (mg/dl)	1.6 ± 2.2	2.2 ± 2.6	
N. of tender joints	7.2 ± 9.1		
N. of swollen joints	5.4 ± 3.7		
Pain VAS	4.75 ± 6.69		
GH	4.6± 2.3		
DAS44	2.41 ± 16.1		
PASI	3.4 ± 2.1	14.6 ± 7.2	
ACPA positivity	0 (0)		
IgM/IgA-RF positivity	0 (0)		
csDMARDs (N/%)	14 (43)	22 (91.6)	
bDMARDs (N/%)	4 (12)	15 (62.5)	

*PsA, Psoriatic Arthritis; Pso, Psoriasis; OA, Osteoarthritis; DAS44, Disease Activity Score 44; ESR, erythrocyte sedimentation rate; CRP, C-reactive protein; ACPA, Anti-Citrullinated Peptide Antibodies; RF, Rheumatoid Factor; VAS, visual analoghe scale; GH, global health; csDMARDs, conventional synthetic Disease-modifying antirheumatic drugs; bDMARDs, biological Disease-modifying antirheumatic drugs*.

Age- and sex-matched healthy donors (HD; *N* = 14) served as normal control (none of them exhibited psoriatic skin or joint symptoms) and were obtained from Blood Center of the Policlinico Umberto I, Roma. Age- and sex-matched OA patients (*N* = 12) served as disease control (Table 1).

The study was carried out according to the Declaration of Helsinki and conducted in accordance with the International Conference on Harmonisation Good Clinical Practice Guidelines. The study protocols were approved by ethic committee of the University of Rome Tor Vergata, Catholic University of the Sacred Hearth of Rome and Sapienza University of Rome. All patients provided written informed consent before participating in any study-related activities.

### Reagents and carbamylation

LL37 peptide was purchased from Proteogenix (F); citrullinated-LL37 (LL37cit), citrullinated at all five arginin position, was from Anawa. Carbamylation of LL37 (LL37carb) was obtained by incubating LL37 with 1M potassium cyanate (Sigma Aldrich) at 4°C for 3 days, followed by extensive dialysis against PBS as described (54). To verify the carbamylation reaction, a dot-blot assay was performed. Briefly, LL37carb were spotted onto a nitrocellulose membrane (GE Healthcare Life Sciences). After blocking in PBS containing 0.05% Tween 20 (PBS Tween) and 5% non-fat dry milk, the membrane was incubated with a polyclonal anti-carbamyl-lysine antibody (Cell Biolabs, Inc., San Diego, CA, USA) and anti-LL37, O/N at 4°C. The next day, after washings with PBS Tween, peroxidase-conjugated goat anti-rabbit IgG (BioRad Laboratories, Richmond, CA, USA) were used as second antibodies and the reactions were developed with 3-3′ diaminobenzidine (Sigma Aldrich).

Antibodies against LL37, (Mab137), was provided by the Antibody Facility of University of Geneva (CMU; CH). Rabbit polyclonal anti-LL37 was from Innovagen. Both were used for immunofluorescence staining in parallel with appropriate control antibodies.

### Human samples

Synovial fluids (SF) were collected from active knee PsA and age- and sex-matched patients with knee osteoarthritis (OA). Exclusion criteria for SF analysis were local intra-articular corticosteroid injection within 5 weeks before SF aspiration. SF was collected via joint aspiration in association with therapeutic arthrocentesis. Approximately 2–4 ml of SF was collected in sodium heparin-coated Vacutainer^TM^ tubes (Becton-Dickinson); samples contaminated with blood were discarded. Immediately after collection, samples were centrifuged at 1,000g for 15 min at 4°C, and the resulting supernatants were stored at −80°C. Plasma from PsA, psoriasis and HD subjects were collected following standard protocol and stored at −80°C. Synovial tissues were collected from Consecutive patients fulfilling the classification criteria for Psoriatic Arthritis [PsA; (55)] undergoing ultrasound guided synovial minimally invasive tissue biopsy following the published protocol using a 14G needle (Precisa 1410-HS Hospital Service Spa, Italy) (56). Once collected ST specimens were fixed in 10% neutral-buffered formalin and embedded in paraffin for Immunohistochemistry (IHC) or immunofluorescence staining.

### Immunohistochemistry (IHC)

Synovial tissue (ST) sections were stained with IgG1 mouse anti-human monoclonal antibody for CD20 (clone L26; at 1.2 μg/ml) or IgG1 mouse anti-human monoclonal antibody for CD3 (clone LN 10; at 1.0 μg/ml) or IgG2a mouse anti-human monoclonal antibody for CD21 (clone 2G9; at 0.34 μg/ml), IgG1 mouse anti-human monoclonal antibody for CD23 (clone 1B12; at 1 μg/ml), IgG1 mouse anti-human monoclonal antibody for Ki67 (clone K2; at 1.2 μg/ml), or IgG2b mouse anti-human monoclonal antibody for Bcl6 (clone LN22; at 1 μg/ml) (all from Leica Biosystem, Newcastle, UK) by immunostainer BOND MAX III (Leica, Newcastle, UK). IHC for CD20, CD3, CD21, CD23, Ki67, and Bcl6 was performed as follows: 3-μm-thick sections were prepared from formalin-fixed paraffin-embedded tissue blocks and were dried in a 60°C oven for 30 min. The sections were placed in a Bond Max Automated Immunohistochemistry Vision Biosystem (Leica Microsystems GmbH, Wetzlar, Germany) according to the following protocol: firstly, tissues were deparaffinized and pre-treated with the Epitope Retrieval Solution 1 (CITRATE buffer) or Solution 2 (EDTA-buffer) at 98°C for 10 min according to the manufacturer's instructions. After washing, peroxidase blocking was carried out for 10 min using the Bond Polymer Refine Detection Kit DC9800 (Leica Microsystems GmbH). Tissues were again washed and then incubated with the primary antibody for 30 min. Subsequently, tissues were incubated with polymer for 10 min and developed with DAB-Chromogen and finally counterstained with hematoxylin (57).

### Confocal microscopy

Three-μm-thick sections in paraffin of human PsA and OA synovia were stained after deparaffination in xilene (5 min, two times), followed by passages in: absolute ethanol (3 min), 95% ethanol in water (3 min), 80% ethanol in water (3 min), 70% ethanol in water, and antigen retrival (5 min at 95°C in 10 mM sodium citrate, pH 6.0). Slides were saturated with blocking buffer (PBS, 0.05% tween 20, 4% BSA) for 1 hour at room temperature. Specimens were stained with a polyclonal rabbit anti-LL37 (Innovagen), rabbit anti-MPO (Abcam), mouse anti-MxA (Novus Bio), monoclonal mouse anti-LL37 (Mab137), polyclonal rabbit anti-human C9 (ATLAS). The following antibodies were used: donkey anti-rabbit IgG AlexaFluor-568 or-647, anti-mouse AlexaFluor-647 and an anti-goat AlexaFluor-488 (Abcam). After washing, slides were mounted in Prolong Gold anti-fade media containing a DNA dye (DAPI) (Molecular Probes). CLSM observations were performed with a Leica TCS SP2 AOBS apparatus, using a 63x/1.40 NA oil objective. Acquisition of images was performed by a Leica confocal software 2.6 (Leica, Germany).

### ELISA

Anti-LL37 antibodies against LL37, LL37cit and LL37carb, were measured by ELISA as previously described (12). Briefly, 96-well flat-bottom plates are coated with 2 μg ml^−1^ of LL37, citrullinated-LL37 or carbamylated-LL37 in carbonate buffer (0.1M NaHCHO_3_, pH 9.6) overnight and washed five times with 0.1% Tween-20 in PBS. This washing buffer was used for washing at all steps. The blocking buffer containing 4% Bovine serum albumin (BSA, Sigma) in PBS was used for at least 1 h (or overnight) to saturate unspecific binding sites. After washing, plasma were diluted 1:100 in PBS 4% BSA followed by 1 hour incubation with a horseradish peroxidase—conjugated goat anti-human IgG (Sigma-Aldrich) diluted 1:5,000 in PBS. Synovial fluids were diluted 1:20. The color was developed with 3,3′, 5,5′-tetramethylbenzidine (TMB) substrate (Sigma-Aldrich). The reaction was stopped by adding 50 μl of 2N H_2_SO_4_ and absorbance determined at 450 nm with a reference wavelength of 540 nm. The cut-off (for both LL37 and related antibodies) was identified by calculating the mean of controls (HD or OA) and by adding 2 standard deviations of the mean: cut-off = mean (HD or OA)+2SD.

To detect IFNα and GM-CSF, plasma and SF were diluted 1:5 and measured by ELISA from MabTech, accordingly to the manufacturer protocol. Plasma and SF content of complement C5a was measured by ELISA from MyBioSource (USA) according to the manufacturer protocol. C9 in plasma and SF was measured by ELISA from Biomatik (USA) according to the manufacturer protocol. LL37 levels of plasma and SF were measured by the Human antibacterial peptide LL37 ELISA Kit (Cusabio, China).

### Statistical analysis

Data were expressed as medians with Interquartile Range (IQR). Differences between median values were determined by two-tailed Mann-Whitney U test (^*^p < 0.05; ^**^p < 0.01; ^***^p < 0.001). Correlation analyses were performed by Spearman rank-correlation test. The cut-off value was determined by using the mean + 2SD (Standard Deviation) of the control subjects (HD or OA). Statistical analysis was performed by using GraphPad Prism software version 6 (San Diego, CA, USA).

## Author contributions

LF conceived the research with RL, directed and supervised the research with the RL, performed staining of tissue biopsies for confocal microscopy, analyzed and interpreted the data, performed statistical analyses, wrote the manuscript. RaP performed most ELISA experiments and helped with statistical analysis. MC, GF, RoP delivered the clinical ethical and patient-related aspect of the project and provided clinical samples (plasma and SF). SA, BT, LP, EG delivered the clinical ethical and patient-related aspect of the project and obtained clinical samples (plasma, SF and ST biopsies). LaB, AE performed immunohistochemistry on ST. EB, AG, LuB, BM delivered the clinical ethical and patient-related aspect of the project and obtained clinical samples of psoriasis patients and patients' data (plasma). SEA provided patients samples and clinical data, performed ELISA. FS acquired and analyzed most confocal microscopy images. MF acquired some confocal images. IP processed blood samples of HD and patients. FRS, CA, FC, GV delivered the clinical ethical and patient-related aspect of the project and obtained clinical samples and patients data (plasma, SF). TC performed protein carbamylation. AC delivered the clinical ethical and patient-related aspect of the project and obtained clinical samples of psoriasis patients and clinical data (plasma). RL conceived the research with LF, directed and supervised the research with the LF, performed analysis of confocal microscopy, performed ELISA, analyzed and interpreted the data, performed statistical analyses, wrote the manuscript.

### Conflict of interest statement

The authors declare that the research was conducted in the absence of any commercial or financial relationships that could be construed as a potential conflict of interest.
